# Markedly prolonged disease course, with breakthrough seizures, in a glioma with an isolated IDH1 mutation

**DOI:** 10.1093/noajnl/vdac004

**Published:** 2022-01-11

**Authors:** Michael Drumm, Jessica W Templer, Matthew Tate, Lawrence Jennings, Craig Horbinski

**Affiliations:** 1 Feinberg School of Medicine, Northwestern University, Chicago, Illinois, USA; 2 Department of Neurology, Northwestern University, Chicago, Illinois, USA; 3 Department of Neurological Surgery, Northwestern University, Chicago, Illinois, USA; 4 Department of Pathology, Northwestern University, Chicago, Illinois, USA

**Keywords:** IDH1 mutation, recurrence, seizure, survival

Gliomas with mutations in isocitrate dehydrogenase 1 or 2 (“IDH^mut^”) are associated with increased risk of seizures, and IDH^mut^ is nearly always found in conjunction with *TP53* and *ATRX* mutations in astrocytomas, or *TERT* promoter mutations and 1p/19q codeletion in oligodendrogliomas. IDH^mut^ gliomas are also less aggressive than their IDH^wt^ counterparts, but still usually recur after treatment and are ultimately fatal; breakthrough seizures are often a harbinger of such recurrences. Here, we report an extremely unusual case where a low-grade glioma only had IDH^mut^ and did not recur or cause breakthrough seizures until 22 years after original diagnosis, without a progression in grade. Yet, seizures manifested at both the original presentation and recurrence. This case raises questions about the nature of IDH^mut^ and its natural course when secondary *TP53*/*ATRX* or *TERT*/1p/19q alterations do not develop.

## Case Report

A 37-year-old female, with no prior medical history, suffered a first-time bilateral tonic-clonic seizure. Imaging at an outside institution revealed a right-sided lesion involving her primary motor strip (images not available). Because of this sensitive location, surgical management was limited to a biopsy, but this biopsy did not yield a firm diagnosis and was deemed non-diagnostic (outside pathology not available). Soon after that biopsy, the patient had her first focal seizure, characterized by clonic movements of the left arm and face. She was initially started on phenobarbital, and carbamazepine was subsequently added due to continued focal motor seizures of the left hand. Later that year, the patient underwent a craniotomy and a second biopsy, again at an outside institution, which generated a pathologic diagnosis of diffuse astrocytoma, WHO grade 2. Adjuvant therapy included 30 fractions of radiation, but no chemotherapy. She was weaned off phenobarbital, continued carbamazepine monotherapy, and remained seizure-free on that regimen for 22 years, with stable MRIs during that entire time.

**Figure 1.**
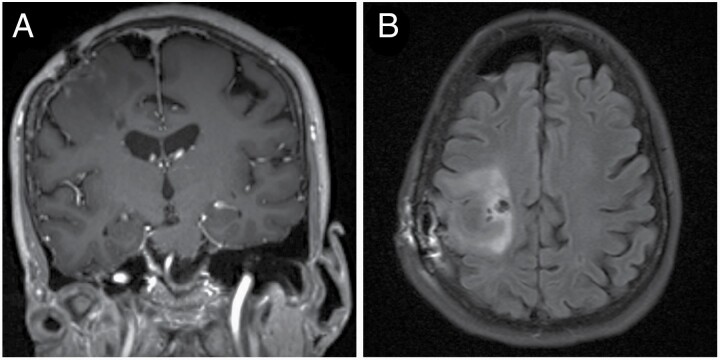
MRI of recurrent IDH^mut^ glioma. (A) Coronal T1 with gadolinium showed an ill-defined lesion in the right frontal perirolandic area with equivocal contrast enhancement. (B) FLAIR in axial plane.

Twenty-two years after her first seizure, the patient (now 59) experienced a focal seizure that was characterized by clonic movements of the left arm and face lasting approximately 15 minutes. An MRI showed an ill-defined lesion in the right frontal perirolandic area with equivocal contrast enhancement (Figure 1). The patient continued to experience focal seizures multiple times a day while still taking carbamazepine, so she was gradually switched to levetiracetam 1000 mg BID. During that transition, she experienced a prolonged focal motor seizure, involving her left arm and face that required hospitalization and intravenous medication to stop. Thus, she was switched back to carbamazepine, but continued to experience one or two focal motor seizures a day. She was then started on clonazepam 1 mg bid, gradually transitioned to clobazam, and her seizures stopped. She has remained seizure-free ever since.

After medication adjustments stabilized her seizures, the patient underwent another biopsy to determine whether the astrocytoma had recurred and progressed.

## Pathology and Molecular Diagnostics

**Figure 2.**
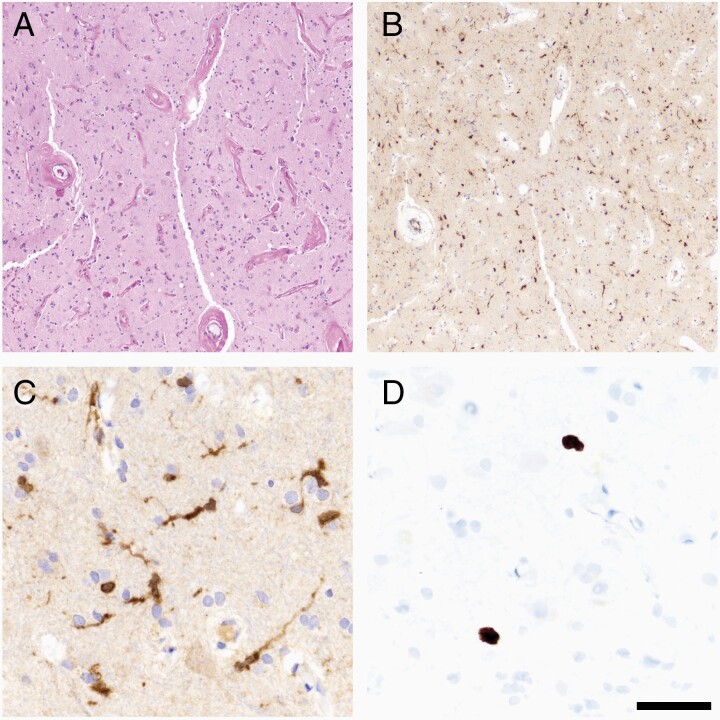
Histopathologic findings. The most recent biopsy from the right motor strip showed mildly hypercellular gray matter with extensive vascular hyalinization (A) Many scattered cells were immunopositive for the canonical IDH1 R132H mutation (B, C). Ki67 proliferation index was low overall, but some cells were positive (D). Scale bar in (D) = 250 microns in (A) and (B), 50 microns in (C) and (D).

The newest biopsy from the right motor strip, 22 years after initial disease presentation, showed mildly hypercellular gray matter with extensive vascular hyalinization, the latter attributable to the remote history of radiotherapy (Figure 2A). Although no obvious glioma cells were identified, immunohistochemistry (IHC) for the canonical isocitrate dehydrogenase 1 (IDH1) R132H mutation revealed unequivocally positive cells with long, ramified processes (Figure 2B and C). Neither p53 nor ATRX IHC showed abnormal accumulation or loss, respectively (not shown). Ki67 IHC showed an overall low Ki67 proliferation index, with only a few scattered cells that were immunopositive (Figure 2D). No necrosis, mitoses, or microvascular proliferation were identified.

Next-generation sequencing of the newest biopsy confirmed the IDH1 R132H mutation with a 7% variant allelic fraction, as well as positive *MGMT* promoter methylation. No other molecular alterations were detected, including no mutations in *ATRX*, *TP53*, or *TERT* promoter, and no copy number alterations on chromosomal microarray.

## Discussion

Molecular diagnostics has revolutionized the workup and classification of central nervous system tumors, allowing for more accuracy than by evaluation of histological findings alone. The original papers describing IDH^mut^ gliomas were published in 2008 and 2009.^1,2^ Now, the classification of adult-type diffuse gliomas is based on the presence or absence of IDH^mut^ (and 1p/19q codeletion).^3^ IDH^mut^, TERT^wt^, 1p/19q-intact gliomas are astrocytomas, and the vast majority of IDH^mut^ astrocytomas contain mutations in *TP53* and/or *ATRX*.^4,5^ In our analysis of 240 IDH^mut^, TERT^wt^, 1p/19q-intact astrocytomas in The Cancer Genome Atlas (TCGA) via GlioVis,^6^ only four (1.7%) were wild-type for both *TP53* and *ATRX*. All four were histologically grade 2, all occurred in 20- to 30-year-old patients (three of whom were female), and all four patients had survived at least 17 months after diagnosis, with no recorded deaths in the database.

IDH^mut^ appears to be the first alteration to occur in IDH^mut^ astrocytomas, with *TP53* and/or *ATRX* arising later.^7,8^ In the current case, the initial tumor was diagnosed by microscopy as a grade 2 astrocytoma, about 10 years before the discovery of IDH^mut^ in gliomas. Although the original tumor tissue was not available, it seems reasonable to assume that IDH^mut^ was present at that time. While IDH^mut^ gliomas are less aggressive compared to IDH^wt^ glioblastomas, IDH^mut^ gliomas are ultimately fatal in the overwhelming majority of cases. Even in the least aggressive subtype—IDH^mut^, 1p/19q-codeleted oligodendrogliomas—survival beyond 15 years is uncommon. In the current case, after 22 years, the recurrent tumor cells were difficult to detect by microscopy, and by molecular testing, those cells only had IDH^mut^. This suggests that IDH^mut^ by itself acts in a relatively indolent manner, and that most cases coming to clinical attention do so because they acquire additional *TP53* and *ATRX* alterations (or *TERT* promoter mutations and 1p/19q codeletion), thereby accelerating tumor proliferation and evolution. Indeed, the long cell processes highlighted by IDH1 R132H IHC (Figure 2C) imply fundamentally more mature tumor cells in this case than are usually encountered in IDH^mut^ astrocytomas. This case also invites speculation as to how often IDH^mut^ occurs in the brain in isolation, and whether most instances might never come to clinical attention if it remains an isolated mutation, especially if it arises in areas less sensitive than the primary motor strip.

This patient’s original tumor, and the first recurrence 22 years later, both manifested as seizures. As the tumor was located in the primary motor strip, those seizures were quite apparent, even though the actual tumor burden (as indicated by variant allelic fraction) was low compared to what is typical for most glioma patients. Our prior work, and that of others, consistently showed that IDH^mut^ gliomas are more likely to present as seizures than IDH^wt^ glioblastomas, even though the latter are more aggressive.^9–12^ Specifically, the D-2-hydroxyglutarate product of IDH^mut^ is secreted by glioma cells and is sufficient to trigger synchronized network bursts in cultured neurons.^9,13^ The association between breakthrough seizures and glioma recurrence is also well known,^14^ although such prolonged intervals between original diagnosis and recurrence are highly unusual, even in IDH^mut^ gliomas.

In summary, this case highlights an extremely rare instance of isolated IDH^mut^ in glioma, with a similarly rare extended patient survival. This also illustrates the fidelity of breakthrough seizures as a marker of glioma recurrence, even in such a prolonged disease course.

## Notes

**Authorship statement**. M.D., J.W.T., L.J., and C.H. prepared the data, text, and figures. M.D., MT, J.W.T., and C.H. edited the manuscript.

**Ethics approval and consent to participate**. Not applicable.

**Consent for publication**. Not applicable.

**Data availability**. Data sharing is not applicable to this article, as no datasets were generated or analyzed during the current study.
